# Using co-design to identify intervention components to address unhealthy dietary and activity behaviours in New Zealand South Asians

**DOI:** 10.1017/jns.2024.48

**Published:** 2024-09-25

**Authors:** Sherly Parackal, Sumera Saeed Akhtar, Sivamanoj Yadav, Rachel Brown

**Affiliations:** 1 Centre for International Health, University of Otago, Dunedin, New Zealand; 2 Department of Human Nutrition, University of Otago, Dunedin, New Zealand

**Keywords:** Co-design methods, Dietary practices, Intervention components, Physical activity, Solutions, South Asians

## Abstract

There is an urgent need to develop sustainable and impactful interventions to mitigate the high risk of diet-related non-communicable diseases (diet-NCDs) in South Asians living in high-income countries. The current study using a co-design methodology aimed to identify community-led intervention components (solutions) to address barriers and enablers of disease-promoting dietary and physical activity behaviours in New Zealand South Asians. Data were collected from South Asian immigrants aged 25–59 years via three focus group discussions (n = 21) and 10 telephone or face-to-face interviews between 2018 and 2019. The thematic analysis resulted in identifying 22 barrier and enabler codes and 12 solution codes which were summarised under five themes. The key solutions (intervention components) to mitigate the identified target behaviours were providing recipes for using local vegetables in South Asian cuisine, information on the nutritional quality of frozen vegetables and canned lentils, simple home gardening techniques, the saturated fat content of dairy foods, interpreting nutrition labels, optimal portion sizes of foods, and framing low-fat messages positively. Similarly, group-based activities with peer support such as walking, cultural dancing and community sports like cricket, football, and tennis were the identified solutions to increase physical activity levels. The identified solutions for health promoting dietary habits and physical activity levels could be part of any targeted multicomponent health promoting programme to reduce the risk of diet-NCDs in South Asian immigrants.

## Graphical abstract

**Figure:**
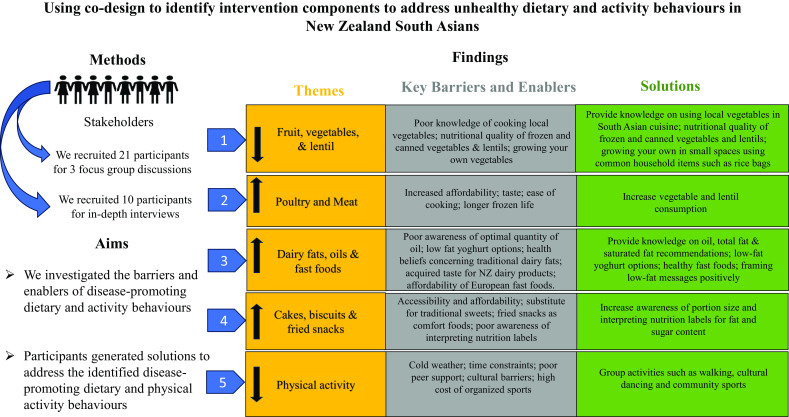

## Introduction

The South Asian diaspora in high-income countries is at high risk for diet-related non-communicable diseases (diet-NCDs).^(1–7)^ The Global Burden of Disease study has highlighted a strong association between suboptimal diets, predominantly low in fibre from whole grain, fruits and vegetables and high in saturated fats, and diet-NCDs.^(8)^ Adopting a ‘western diet’ that is high in saturated fats is a common phenomenon observed among South Asian immigrants^(9)^ and New Zealand South Asians are no exception.^(1,10)^ Among New Zealand South Asians, fruit and vegetable consumption decreased, whilst consumption of meat, poultry, processed meat, potato chips, cakes, pastries, festival foods, takeaways, and alcohol increased as the duration of residence in New Zealand increased.^(10)^ Not surprisingly, body mass index (BMI) also increased as the duration of residence increased.^(10)^ Similar findings have been reported in the Metabolic Syndrome and Atherosclerosis in South Asians Living in America Study (MASALA),^(4)^ which indicate that in contrast to ‘fruits, vegetables, nuts and legumes’ dietary pattern, ‘animal protein’, and ‘fried snacks, sweets and high-fat dairy’ dietary patterns were associated with greater insulin resistance and lower HDL cholesterol.^(4)^ A strong positive association also exists between regular physical activity and adherence to healthy eating habits;^(11)^ nevertheless, levels of physical activity in the South Asian diaspora are inadequate, for example, the New Zealand health survey data findings indicate that only 46% of South Asians aged 15 + years were physically active.^(2)^

A review of dietary changes and their impact on the trajectory of health among South Asian immigrants in Western countries has highlighted the first five years post-migration as a window of opportunity to promote healthier dietary habits to reduce the risk of diet-NCDs in the South Asian diaspora.^(12)^ Hence, designing and implementing culturally appropriate health-promoting interventions to promote healthier dietary habits of South Asian immigrants could prolong their healthy migrant status and reduce the diet-related disease burden. The InnvaDiab-DEPLAN study conducted in Norway on Pakistani immigrants showed that a health-promoting intervention was effective in changing intentions to make their dietary habits healthier and resulted in modest changes in dietary habits^(13)^ with a longer-term maintenance of these changes.^(14)^

Given the uptake of universal health-promoting interventions is much higher among European people in comparison to people of South Asian origin,^(15)^ probably due to recruitment challenges^(16)^ and cultural inappropriateness of the developed intervention,^(17)^ developing culturally appropriate interventions is prudent to address health disparities prevalent in the South Asian diaspora. Moreover, there is substantial evidence that interventions developed using a shared decision-making approach are more beneficial than culturally adapted ones.^(18)^ Co-design is a participatory action research method that has been used to develop culturally appropriate interventions targeting minority populations.^(19,20)^ Studies have shown that using co-design throughout the intervention development process will facilitate tracing significant intervention components to the generated solutions from the co-design research process^(21)^ and being participant centered, co-design research is more likely to avoid resource wastage due to ineffective interventions and reduce the gap between research and practice. In this paper, we report the outcomes of a study that used a co-design approach to identify ethnic specific intervention components to reduce the risk of diet-NCDs in New Zealand South Asians. First, we report the barriers and enablers of disease-promoting dietary and activity behaviours followed by the solutions (intervention components) generated by the participants to address disease-promoting dietary and physical activity behaviours.

## Methods

This study used a community-centric approach, inherent to co-design methodology^(22)^ described previously.^(23)^ Briefly, this involved extensive engagement with the South Asian community in 2017 to obtain community buy-in, a key building block towards addressing health disparities.^(24)^ Three community meetings were held in a culturally appropriate manner and included the attendance of community leaders and cultural food. For this study, stakeholders were consulted at two time points to ensure cultural safety, cultural appropriateness, and cultural integrity were upheld throughout the research process. Adhering to the principles of co-design, which is underpinned by the theoretical framework of participant action research^(25)^ and founded on the principle that the community of interest are the experts,^(26)^ the researchers went beyond just stakeholder consultation and facilitated active participation of the target population in generating solutions (intervention components) for the identified barriers. Using an iterative process first, three focus group discussions (FGDs) followed by ten semi-structured interviews were conducted. All participants provided written informed consent before participation. The FGDs and interviews were audio recorded and conducted by ethnically matched personnel to ensure cultural appropriateness. All procedures performed in this study were in accordance with the ethical standards of the institutional research committee and with the 1964 Helsinki declaration and its later amendments or comparable ethical standards. All participants signed an approved consent form prior to participation. Ethics approval was obtained from the University of Otago Human Ethics Committee (18/205; dated 14/12/2018).

### Study participants and recruitment

The participants for this study were first generation South Asians, i.e. people from India, Sri Lanka, Nepal, Pakistan, Bangladesh, and Afghanistan, aged 25–59 years both ages inclusive, who understood and could communicate in English and were living in Dunedin or Auckland, New Zealand. Participants meeting the study’s inclusion criteria were recruited via advertising through established links of the stakeholders and researchers to various South Asian cultural and religious groups in New Zealand. Data for this study were collected between 2018 and 2019.

### Study design, data collection and analysis

This study used qualitative research methods on a convenient sample of New Zealand South Asians to achieve the study objectives. Participants recruited for the FGDs, and the semi-structured interviews completed a short demographic questionnaire before participation. Three FGDs (∼90 min) with 5–9 participants of different duration of residence (DoR) (≤ 5 years (n = 9); >5<10 years (n = 5) and ≥ ten years (n = 7); total n = 21) in each were conducted to capture the experiences of immigrants of different DoR on changing dietary habits and physical activity patterns post-migration, barriers to adopting healthy dietary habits and physical activity and generating solutions to address unhealthy dietary patterns and physical activity levels. FGDs were commenced by general discussions on post-migration changes in food habits, concerns regarding changing dietary habits and long-term health, sources of health information, and prioritisation of health as migrants in a western country. Following this, findings of a previous study (data collected in 2017)^(10)^ were used as a starting point for more focused discussions. For example, in the previous study, consumption of vegetables had decreased post-migration, with only 15% of males and 36% of females meeting the three-plus-a-day recommendation for vegetable consumption.^(10)^ This was discussed in the three FGDs, and participants were asked about the barriers they faced in consuming adequate vegetables and the solutions they perceived from their own experiences and knowledge that would be effective to increase vegetable consumption. Following the FGDs, ten semi-structured interviews (45–60 min) were conducted face-to-face and via telephone with the target population aiming to triangulate the FGDs findings. The topic list for the interviews was generated from the findings of FGD data.

SMY and SMP transcribed all the recordings verbatim, and the transcribed data were thematically analysed. The transcripts and field notes were continually re-read to become familiar with the data and identify relevant codes to the research questions. Transcripts were initially manually coded by one researcher (SMY), and the codes were used to generate themes and subthemes following an inductive thematic analysis approach. The analysis begins with familiarisation, generating initial codes, searching for themes, reviewing the themes, defining and naming themes, and producing the report.^(27)^ Microsoft Excel^®^ was used to organise the transcripts so that the initial coding process could be completed. All the transcripts were also imported and analysed into NVivo 12 Plus by SSA, a qualitative analytic software program for data storage, coding and retrieval, and text analysis.^(28)^ Coded transcripts were subsequently compared, and areas of disagreement were resolved through regular debriefing meetings held between SMY, SSA, and SMP to verify the codes and reach a consensus. This iterative process permitted reviewers to closely examine significant issues raised in the focus groups and interviews to identify specific themes, subthemes, and subtopics. The coding guide was revised to include additional themes due to these consensus meetings. Once coded, all transcripts were merged, and frequently used codes were sorted into categories. Finally, one researcher coded and analysed all transcripts (SMY). Through a final round of selective coding, the study team reviewed significant quotes within each code category, aggregated across all transcripts, to facilitate the identification of significant themes. All study authors then met to agree on the significance of themes, subthemes, and subtopics and to select and agree upon quotations to illustrate themes. Participant quotations throughout the manuscript are italicised, some were edited for length and clarity and indicate whether the participant attended the FGD or in-depth interview along with the associated unique identifier number.

## Results

The majority of South Asians who participated in the FGDs and the interviews were early immigrants (≤5 years in New Zealand), women (>60%), from India (>60%), identified as “Hindu” (>55%), had a tertiary level qualification (>90%), were employed (>80%), were from a middle to high-income household (NZ$ 20,000+; 33–60%), were aged below 35 years (61% in FGD and 80% in Interview), with a mean age of 38.3 (SDM10.9) years in the FGD and 33.6 (SDM 3.6) years in the interview (Table 1). The thematic analysis resulted in 22 barrier and enabler codes and 12 solution codes summarised under five themes, which are discussed below. Detailed illustrative quotes of the barriers and enablers of unhealthy behaviours (Suppl Table 1), and the solutions to mitigate these behaviours (Suppl Table 2) are provided as supplementary data.

**Table 1.** Demographic characters of the focus group discussions (n = 21)* and in-depth interview participants (n = 10)

Demographic characteristics	Focus group discussion	Interview
Mean age (SD)	38.3 (10.9) years	33.6 (3.6) years
*Sex (n (%))*		
Men	8 (44)	4 (40)
Women	10 (66)	6 (60)
*Country of origin (n (%))*		
India	12 (67)	6 (60)
Pakistan	1 (5.5)	1 (10)
Sri Lanka	2 (11)	1 (10)
Nepal	2 (11)	1 (10)
Bangladesh	1 (5.5)	1 (10)
*Religion (n (%))*		
Hindu	10 (55)	7 (70)
Muslim	3 (17)	1 (10)
Christian	2 (11)	2 (20)
Buddhist/other	3 (17)	–
*Education (n (%))*		
Completed Tertiary	18 (100)	9 (90)
Some Tertiary	–	1 (10)
*Employment (n (%))*		
Employed	10 (56)	8 (80)
Not Employed	8 (44)	2 (20)
*Household income per year (n (%))*		
Less than $NZ20,000	3 (17)	2 (20)
$NZ20,000–50,000	6 (33)	6 (60)
More than $NZ50,000	9 (50)	2 (20)
*Duration of residence in NZ (n (%))*		
Early migrant (≤ 5 years)	13 (72)	9 (90)
Established migrant (>5 years)	5 (28)	1 (10)

* Demographic data was missing for n=3.

### Theme 1: inadequate consumption of fruits, vegetables, and lentils

The critical barriers to adequate consumption of fruit and vegetables primarily were the high cost of fruit and vegetables, lack of availability of fresh traditional vegetables, and difficulty in developing a taste for new varieties of fruit and vegetables in New Zealand.

*In 9: New Zealand does have vegetables, but the price of the vegetables are (is) humongous. …., like obviously we all go through a budget in a week and that is the hardest time that when we know that half cauliflower is like three or four dollars or an eggplant is like five dollars.*

Participants felt accepting what was locally available and trying new fruits and vegetables was the solution to increasing fruit and vegetable consumption. This was also true for increasing lentil consumption. Lack of knowledge about cooking locally available vegetables was also identified as a barrier to adequate vegetable consumption. Substituting local vegetables in South Asian cuisine was identified as a solution with participants sharing their experiences, for example, substituting Kale for traditional greens in cooking Pulau (Pilaf).

*FGDP7: we don’t have (the) kind of greens here (which we had in India), tried to cook the available greens in similar way, tasted really good. Kale tasted really good, I made pulau and aroma is quite good.*

Another suggested solution for increasing vegetable consumption was to use frozen or canned traditional vegetables available from local ethnic stores. However, many participants were skeptical of the nutritional quality of both frozen and canned vegetables. The lack of variety of traditional vegetables also had a follow-on effect on less rice consumption, which was replaced by more pasta and noodle consumption, with little or no vegetables.

*In 3: lack of variety is the main reason; you don’t want to eat the same thing (vegetables) all week … if you had to get seven varieties of vegetable shares for seven days, then I would probably have eaten more rice. And if you eat rice, then we can do vegetables as well…. In pasta, there are no vegetables. Usually, pasta is some meat, some onions and some garlic.*

Participants also suggested growing some vegetables would be a solution to overcome the high cost and lack of availability of some traditional vegetables, especially herbs and greens. However, many participants raised the issue of lack of knowledge and space for gardening as barriers to having a vegetable garden. The suggested solution to overcome this barrier was providing information on how to grow their own using containers, especially re-using common household items such as rice bags.

### Theme 2: increased consumption of poultry and meat

Most participants reported that their meat intake had increased post-migration due to a change in lifestyle in New Zealand. Enablers of increased consumption of poultry and meat were affordability, taste, ease of cooking, and longer frozen life. Participants attributed the increase in meat consumption to a decrease in consumption of vegetables and lentils.

*In7: I think it’s changed a lot. I would say it’s worse because my diet pattern back home was more of vegetarian food. Non-vegetarian was very rare. And even if it was non-vegetarian, we used to have a lot of variety of vegetables every day. But here, it’s very much limited. Even considering my diet, I’ve started eating more non-vegetarian, and the consumption of vegetables has dramatically reduced.*

### Theme 3: increased consumption of dairy fats, oils and fast foods

Generally, oil consumption increased post-migration, and most participants indicated that they did not keep track of how much oil they consumed daily, weekly, or monthly. The choice of oil varieties had changed for participants after shifting to New Zealand, primarily due to cost factors or based on the perception that certain oils were healthier.

*In4: Yeah, so I don’t have to be too conscious that I’m using extra, you know, more oil or anything so I can use it (olive oil). Otherwise, I would feel guilty that oil is not good so I try and use extra virgin olive oil.*

Consumption of coconut oil had increased significantly among those from Southern India and Sri Lanka, particularly due to recent surge in social media promotion. Participants perceived that coconut oil was healthy, although some were uncertain whether it was good for their health.

*In10: I have started using coconut oil recently because it’s good for you. And I’ve got even a big bucket of coconut oil, so I use it … but I still don’t know if… I don’t know if it’s good.*

Most participants maintained their consumption of traditional dairy foods such as ghee and yoghurt and, in addition, consumed more ice-creams and cheese.

*FGD P15: I am from Punjab (India or Pakistan). So I love milk products and cut them (reduced consumption). When I started (living in New Zealand), I ate cheese a lot. I used to buy an entire block, and then I’ll finish it in two weeks.*

Strong belief in the health benefits of ghee was an enabler for the continued consumption of ghee. Enablers of increased ice-cream consumption, cheese and butter were the numerous varieties of ice creams, easy access and affordability, and the overall high quality of dairy products in New Zealand. Yoghurt consumption had also increased among most participants, nevertheless participants were unsure of low-fat options of yoghurt.

*In 6: I generally buy a natural yoghurt, so I don’t know whether it has a high or low fat.*

The consumption of Indian fast-food decreased among participants after moving to New Zealand; in contrast, their consumption of other fast foods such as fish and chips, KFC, Dominoes and Chinese increased. Dietary acculturation played an essential role in the increased consumption of western fast foods such as fried chicken and pizzas, primarily due to their affordability.

*In9: When you come to a new country, you think about the cost maximization and minimization. And that’s the reason we focus on KFC and McDonald’s because of the price, the reasonable price.*

Solutions suggested by participants to reduce consumption of high-fat dairy foods and oils were educating people on the optimal portion size of dairy foods and oils, framing low-fat messages positively, providing more information on the saturated fat content of foods and general information on recommended levels of fat intake.

*In3: If you say cheese is bad for me, that will give a negative effect on me. But if you say like, a one slice of cheese or a gram or whatever you need (recommended), you can say like, okay, if you eat that, that’s good for health. But if you increase that, (that might not be good for your health). If you say that then that’s good for me. The word (framing the message positively) used for the thing (dairy food) that we are consuming, and we feel like that is good.*

### Theme 4: increased consumption of cakes, biscuits, chips, and snacks

Most participants reported that they had increased their consumption of cakes and biscuits in New Zealand more as a substitute for traditional sweets. Dietary acculturation increased consumption of cakes, biscuits, chips, and snacks. Increased accessibility and affordability also enabled increased consumption of cakes, chocolate biscuits, butter cookies, and chips. Some participants reported regular consumption of salty fried foods such as crisps/potato chips, with some describing these as comfort foods.

*In 9: Yeah, because I couldn’t get used to this country’s food habits and didn’t know what to do. So, those (chips) were my comfort foods to make me feel happy and good, but then I realised that that was increasing my weight.*

Many participants were unaware of the health impact of consuming deep-fried and highly salted snacks regularly and did not read the label for nutritional information. Those participants who read the nutrition labels did so to know if the product contained animal fats (vegetarians), was Halal (Muslims), or contained preservatives.

*FGD P9: So, when I look at nutrition labels, it was more of what I would be concerned about the preservatives …. everything that’s happening to us, our health and environmental issues, is because of all these preservatives which are going in our body.*

Solutions identified by participants to address excessive consumption of high-fat, high-sugar, and salty snacks were to increase awareness and knowledge on reading and interpreting nutritional labels on packed foods and optimal portion sizes.

*FGDP4: yeah that kind of information (nutrition labels) is really important. Especially because people from India for example, based on my experience, we get everything from family and our mother would take care of us. So we do not know about nutrients and what a serving should look like.*

### Theme 5: lack of adequate physical activity

Many participants felt that a lack of physical activity among South Asian immigrants was an important issue to be addressed. Although participants were aware of the importance of staying active, they felt that they were not being active enough. Lack of knowledge of the benefits of physical activity was not a barrier to being physically active in New Zealand. However, the change in the living conditions in New Zealand was a barrier.

*In7: Yes, I know about that (activity and health), but where will I fit it? I was, I would say, more physically active because I used to walk to the bus stop and even travel by bus. We used to do physical activity (back home). There was more walking and climbing and stuff like that. But here, lazy lifestyle for me.*

Key barriers faced were frequent cold and wet weather, time constraints due to prioritising work and family responsibilities, lack of peer support, cultural barriers, and the high cost of organised sports.

*In6: My major concern here, only one thing I find it is the cold weather, which is I’m still getting adjusted to the weather. So, as it’s very cold, I try my best not to step out walking.*

Participants indicated that having peer support would encourage them to be regularly active via walking, trekking, cultural activities such as dancing, and knowledge about community sports and walking tracks. Preferred activities included walking, cultural dancing, and community sports like cricket, football and tennis.

*In 4: I want company. I think that’s my problem. I think (walking) anywhere is fine as long as I have some company….Put together a walking group or something like that. you know, I mean having our community would be somewhat better because we can just enjoy more fun talks (as we walk).*

## Discussion

In this paper, we discuss in detail the barriers and enablers of unhealthy dietary and physical activity behaviours in New Zealand South Asians who have a high risk for diet-NCDs such as diabetes. One risk factor for diet-NCDs consistently reported in national and international studies is poor fruit and vegetable intake among South Asians living in Western countries.^(1,3,8,10)^ Hence, understanding the modifiable factors to improve fruit and vegetable intake is critical to reduce the burden of diet-NCDs in this population. The finding that the high cost of fruit and vegetables in New Zealand was a deterrent to adequate consumption was similar to an Australian study on the health behaviours of South Asian immigrants^(29)^ and is likely to impact similarly for other population groups in New Zealand and elsewhere. However, key modifiable barriers identified for adequate fruit and vegetable consumption specifically among New Zealand South Asians in the current study were the poor availability of fresh traditional vegetables, lack of knowledge about cooking locally available vegetables in South Asian cuisine, and skepticism on the nutritional quality of frozen and canned vegetables and lentils. Among other factors, such as affordability, increased meat and poultry consumption was reported as a replacement for the lack of traditional vegetables. In a study of Pakistani migrant women in Oslo, Norway, increased affordability of meat and poultry resulted in daily meat consumption in contrast to eating meat once or twice a week while they lived in Pakistan.^(30,31)^

In the current study, South Asians were more concerned about the quality of oil rather than the quantity of oil consumed, and overall oil consumption and dairy fat consumption increased post-migration. The finding that fat consumption increased post-migration is consistent with other studies on South Asians in Europe^(30,31)^ and North America.^(32)^ The current study indicates that the increase in total fat consumption was primarily due to the consumption of ice cream and cheese in addition to traditional fat sources. In the Oslo study, Pakistani South Asians increased their oil intake, whereas Sri Lankan South Asians increased their butter and oil consumption.^(30,31)^ A previous New Zealand study also reported increased fast food (takeaway) consumption among South Asian males.^(1)^ The current study indicates that the types of fast food consumed by South Asians, which were predominantly high-fat options such as Kentucky Fried Chicken, Pizzas, and deep-fried fish and chips, are significant contributors to the increased fat consumption observed among South Asian immigrants in Western countries. Similarly, the increased consumption of cakes, biscuits, potato chips, and other fried snacks observed in the current study was primarily to replace traditional sweets and snacks. In the MASALA study, those with strong cultural beliefs were more likely to consume a diet high in fried snacks, sweets, and high-fat dairy.^(32)^ The findings of the current study, for example, “ghee” was considered healthy, the excessive use of coconut oil and that participants were not concerned about the quantity of oil or the fat content in yoghurt, all resonate with the findings of the MASALA study, which reported a strong association between cultural beliefs and high-fat consumption.

The co-design methodology adopted in the current study enabled the generation of solutions as intervention components to address unhealthy dietary and physical activity behaviours. The participants identified several solutions to address detrimental dietary behaviours. One of the solutions generated for improving vegetable consumption was providing tips and techniques for home gardening. Home gardening has been shown to be perceived as an effective measure to increase consumption of fresh home grown produce, reduce fast-food consumption, increase motivation to be healthy, increase physical activity, mental health, and the ability to better manage stress by an ethnic minority population of low-socioeconomic status in the US.^(33)^ Similar associations have been shown between fruit and vegetable consumption and being involved in community gardening.^(34)^ Community gardeners significantly increased their intake of total vegetables by 0.63 servings (P = 0.047) and garden vegetables by 0.67 servings (P = 0.02) in a randomised controlled trial in the US.^(34)^ Recipes to substitute locally available vegetables in South Asian cuisine were another solution generated for increasing vegetable consumption. Demonstration of recipes via culinary instruction maybe one method of achieving this. Community-based nutrition education that included culinary instruction has shown promise in changing nutrition behaviours and improving cardiometabolic biomarkers in a sample of low socio-economic ethnic minority participants.^(35)^ Educating people on the optimal portion size of dairy foods and oils, framing low-fat messages positively, providing more information on the saturated fat content of foods, and general information on recommended levels of fat intake were solutions suggested for reducing consumption of high-fat dairy foods and oils. Solutions identified by participants to address excessive consumption of high-fat, high sugar, and salty foods were to increase awareness and knowledge on optimal portion sizes and read and interpret nutritional labels on packaged foods. Studies have demonstrated that, if empowered with the correct knowledge regarding diet, South Asians can bypass cultural obligations and fulfil healthier choices, highlighting the need for greater education in the community.^(36–40)^ Several reviews have indicated that culturally adapted or tailored interventions have the potential to improve nutrition-related outcomes including reduced portion sizes and saturated fat.^(41,42)^ These analyses suggests that co-creation practices hold potential for tailoring nutrition interventions in collaboration with Indigenous and ethnic minority populations.

Increasing physical activity was considered necessary for South Asians, albeit a lack of knowledge of the health benefits of physical activity was not a barrier among the South Asian participants of the current study. A review of healthy lifestyle changes in minority ethnic populations in the UK found that knowledge about the positive impact of physical activity on health was poor among South Asian communities in the studies reviewed.^(43,44)^ Previous studies among South Asian participants showed that whilst they were generally aware of the health benefits associated with physical activity,^(40,45–47)^ there was limited understanding of the actual levels of physical activity required to gain health benefits.^(40,45,47)^ Studies on South Asians have indicated that the barriers to increased physical activity include prioritising work over physical activity to provide for the family^(29,43,44,48,49)^ the need to serve and eat traditional foods, and the different perceptions of healthy body weight.^(44)^ In the current study, in addition to prioritising work over allocating time for physical activity, other barriers included the changed living conditions post-migration, cold weather, and the cost of organised sports. These findings are similar to those reported by a U.S. study, where significant barriers to adequate physical activity among South Asians included changes in post-migration living conditions, the cold and wet weather, and the financial cost of being engaged in organised sporting events.^(50)^ Other studies have also identified cultural barriers^(29,43,50)^ and cultural beliefs^(29)^ on physical activity to be significant barriers to adequate physical activity among the South Asian diaspora.

A key solution suggested by participants for increasing physical activity was peer support. Peer-led interventions to increase physical activity levels have been shown to be as effective as those led by professional physical activity providers across several population groups.^(51)^ Participants felt that having peer support or doing physical activity with other South Asians would encourage them to be regularly active. Kalavar *et al.* found that lack of peer support was a key barrier to increasing physical activity among South Asians in the U.S.^(48)^ The finding from the current study resonates well with other studies investigating enablers of physical activity in South Asians. Nisar *et al.* found peer modelling was a motivating enabler for walking, cycling, and group sports activities.^(29)^ Other studies have found that group physical activity sessions had health and social benefits for South Asians.^(42)^ A meta-analysis of the effectiveness of interventions to promote physical activity in the general population has found group delivered interventions to be more effective in changing behaviour compared to individually delivered interventions.^(52)^ More specifically, walking in groups was found to be efficacious at increasing physical activity across different populations in different countries.^(53)^ Preferred activities identified in the current study for increasing physical activity were walking, cultural dancing, and community sports like cricket, football, and tennis, which are all group based. A feasibility study among South Asian women in the Netherlands has shown some promising results in increasing physical activity levels by implementing cultural dancing (Bollywood) as a form of physical activity.^(54)^ A study among South Asians in Scotland found that physical activity that provided a platform for socialising and enjoyment was a key motivator for engaging in physical activity.^(46)^

The current study is unique in identifying participant generated ethnic-specific intervention components to address established risk factors of diet-NCDs. The generated solutions have the potential to develop a multicomponent intervention to reduce diet-NCDs in South Asians. Multicomponent interventions have been shown to have a higher level of effectiveness in achieving the targeted behaviours.^(55)^ The key strengths of the current study were the strong community buy-in, which resulted in a high level of participation by the South Asian community as stakeholders and participants. SP, SA, and SMY are all South Asian, are multilingual, and have extensive connections with the South Asian community in New Zealand of various faiths and socio-economic backgrounds. Hence, common limitations such as cultural sensitivity and language barriers did not hinder recruitment, data collection, or analysis. Although the FGDs and interviews were all conducted in English, most participants expressed themselves in their mother tongue frequently during the FGDs and interviews. An inherent limitation of the current study is the generalisability of the findings to the broader South Asian communities in New Zealand and elsewhere. The participants of the current study were highly educated; hence, the findings of this study may not be the same for South Asian immigrants with low education levels. This was evident in the data regarding knowledge about the benefits of physical activity on health, which was high among the participants of this study. Nevertheless, this knowledge did not necessarily translate into behaviour, and the barriers to adequate physical activity were similar to those found in other studies.^(26,40,41,45,46)^ Moreover, the depth of enquiry that was undertaken generated information broadly transferable to the South Asian diaspora worldwide to enable a targeted health promotion initiative for prolonging the healthy migrant status of South Asians and reducing the burden of diet-related chronic diseases in this population. Currently, the evidence for lifestyle interventions developed using non co-design methods to reduce the risk of diet-NCDs in South Asians is at the best, moderate.^(56)^ The question whether interventions developed using co-design are more effective in reducing the risk of diet-NCDs in the South Asian diaspora is yet to be answered. The next phase of this project is to develop a co-designed, multicomponent, health promoting intervention incorporating the intervention components from this study and test its effectiveness in promoting healthier diet and physical activity levels in New Zealand South Asians.

## Conclusions

The current study identified key modifiable barriers for low consumption of vegetables and fruits, high consumption of fats, unhealthy takeaway foods, and inadequate physical activity among South Asian immigrants in New Zealand. The identified solutions to mitigate these barriers were providing information on simple home gardening techniques, the nutritional quality of frozen vegetables and canned lentils, recipes to substitute local vegetables in South Asian cuisine, educating people on the optimal portion size of dairy foods and oils, framing low-fat messages positively, providing more information on the saturated fat content of foods, knowledge on optimal portion sizes and reading, and interpreting nutritional labels on packaged foods. Similarly, group-based activities with peer support such as walking, cultural dancing and community sports like cricket, football, and tennis were the identified solutions to increase physical activity levels in South Asians. The identified solutions for health promoting dietary habits and physical activity levels contribute to creating a multicomponent intervention that is achievable and can be part of any targeted health promoting programmes to reduce the risk of diet-NCDs in South Asian immigrants.

## Supporting information

Parackal et al. supplementary material Parackal et al. supplementary material

## Abbreviations

**diet-NCDs:** diet-related non-communicable diseases; **FGDs:** focus group discussions; **DoR:** duration of residence; BMI: body mass index; **MASALA:** Metabolic Syndrome and Atherosclerosis in South Asians Living in America Study.

## Supplementary material

The supplementary material for this article can be found at https://doi.org/10.1017/jns.2024.48
