# Supplementary material for: Using co-design to identify intervention components to address unhealthy dietary and activity behaviours in New Zealand South Asians
Source: J Nutr Sci. 2024 Sep 25;13:e47. doi: 10.1017/jns.2024.48 (PMC11428100; doi:10.1017/jns.2024.48)
Supplement: Parackal et al. supplementary material [file S204867902400048Xsup001.docx]

Supplementary Table 1: Barriers of and Enablers for unhealthy dietary and activity behaviours

| **Theme** | **Codes associated with the theme** | **Illustrative Quotes** |
| --- | --- | --- |
| 1. Inadequate consumption of fruits, vegetables, and lentils | High cost of fruit, vegetables, and lentils: **Barrier** | 1. FGD P9: They're (fruit) very expensive, so whenever I want to eat a watermelon, one slice costs me around five to six dollars, which in India, I can eat five to six watermelons at that much rate. 2. In 9:…. New Zealand does have vegetables, but the price of the vegetables are (is) humongous. ….., like obviously we all go through a budget in a week and that is the hardest time that when we know that half cauliflower is like three or four dollars or an eggplant is like five dollars. |
|  | Lack of availability of fresh traditional vegetables: **Barrier** | 1. FGD P1: In Bangladesh, there are so many vegetables, plenty of vegetables. When I come here, I find a couple of vegetables like this (that here). We all know what kind of vegetables they are. 2. FGD P16: When it come(s) to winter we only have few choices only like, like cabbage, cauliflower. We get exhaust(ed) with the same vegetables having every time. 3. In 2: Mostly, I feel that we don't get much (many) varieties here, to cook our Indian food here, so we are skipping most of the vegetables, what we used to get in India. It's like, we're just using same mushroom. In a week, I'll tell you, we eat only mushroom, cabbage, cauliflower, and two days, lentils. |
|  | Difficulty in developing a taste for new variety of fruit and vegetables: **Barrier** | 1. FGD P10: I tried at least feijoas, dragon fruit, and passion fruit. You have never heard of those fruits but try to taste them. I frankly didn't like any of these: Why are they so tangy or bitter? 2. In 6: New varieties, new tastes getting used to. …we don't get it (traditional vegetables) and I think even if we get it, I don't know, it doesn't taste exactly the way how it used to taste back home in India. |
|  | Lack of knowledge about cooking local vegetables: **Barrier** | 1. FGDP2: we have plenty of vegetables also (alternatives). I try to grow silver beets, strawberries, kale… don’t know how to cook and consume. |
|  | Skepticism about the nutritional quality of canned and frozen traditional vegetables: **Barrier** | 1. FGD P9: Until now I heard I feel that canned food & frozen are not healthy. 2. In 8: When it comes to canned food, whether it's canned tomato, canned beans or any other canned food, I'm sure they must have added some preservatives in it. And you don't know how healthy those preservatives for us are to consume. 3. In 9: Because usually, I find it cooking time is also not appropriate (adequate) for us and there are (is) lack of vegetables as well that what we usually have we always seen in our country it's more of fresh, but here, it's not that kind of food which we see. So, it's more of frozen, frozen stops (us from eating) some more (vegetables). So, that (our food) doesn't contain any nutritious diet and the (dietary) habit changes. |
|  | Lack of knowledge about gardening and space for gardening: **Barrier** | 1. In1: I think simple vegetables like chillies or something which we can grow near our kitchen garden would be helpful. Because sometimes we try to grow, but we don't know how to exactly do that (grow), so it don't turn out to be so good, so things just grow worse. 2. In6: Yeah, for now, in my apartment it's not possible but once I move to a bigger space where I have (space), yeah, I've been thinking if I can grow my own vegetables …., maybe whichever is quite easy. sometimes I feel like tomatoes… or mint leaves. 3. In10: Where I am right now, there's not much space for gardening. So, I've got some pots and started growing capsicum and chilies. Just, that's all I could grow in a small pot a lot of vegetables can't grow in a small pot. |
| **2. Increased consumption of poultry and meat** | More affordable and tastier than vegetables: **Enabler** | 1. FGDP2: Compared to vegetable here meat I think it’s cheaper I think that's the reason you tend to buy more meat than vegetables. 2. FGDP6: For taste and price people tend to buy meat (more) than vegetables. 3. FGDP16: …... Mushrooms and chicken if you compare, mushrooms are expensive. |
|  | Ease of cooking and storage: **Enabler** | 18) FGD P1: I think it's easy to cook, its available, and … you don't have to do much work …buy the chicken or beef just cook and it stays longer as well…The thing is you can store meat for a long time. |
| **3. Increased consumption of dairy fats, oils and European takeaways** | Strong beliefs on health benefits of Ghee, coconut oil and olive oil: **Enabler** | 19) FGDP9: .. the oils, which you see around sunflower oil and all this, the nutritional value in ghee is much more….I use ghee for everything I cook. … but for salads, I use olive oils.  20)FGDP13: I think ghee is a very important. your body needs it. Instead of going on (eating) margarine. Yes, they (margarine) are cheaper. You don't have health benefits. Whereas if you're using those things (Ghee and Butter) in moderate, it is good for you.  21) In9: I use ghee mostly, but again, ghee is really good for health as well. I use ghee for lentils. Sometimes I make ghee rice. In maybe two months, I have one kg (of ghee). |
|  | Availability of variety of dairy foods: **Enabler** | 22) FGDP2: There are different varieties of ice-creams available here back in India we don’t. Whenever you go to supermarkets, we have like different varieties (which) they are marketing also, …. mixing (ice cream) with cookies, chocolates and different varieties  23) In2: When I came initially, in 2017, in New Zealand, all of a sudden, I have come from India. So, obviously, I used to get attracted to the ice creams and chocolates, because there is so many different varieties here. I consumed a lot initially, when I came here…but out of my craving, I just like to eat. |
|  | Increased accessibility and affordability: **Enabler** | 24) In2: Yeah, because in India, I used to not eat ice creams regularly, but it's easily accessible here. When I go to (buy) groceries, the first thing you'll see is ice creams, different flavors and different varieties, which will tempt the customers  25)In 8: We never used to have so much of ice creams in India, but here, you get a one kg or two kg of box for $4, and it's readily available in the supermarket. |
|  | High quality dairy foods and oils: **Enabler** | 26) FGDP3: People talk about coconut oil and I do know when it was popular everyone wanted coconut oil and put it on toast and have it.  27) In1: I am aware (bad effects of butter), but the only thing is it is easily available, we like butter, but it was not too good quality over there (home country), but here I think the flavour is so awesome and so good. But I think we are having it more here. So yeah, the cream, cheese, butter and yoghurt, yeah we are having it more and the flavored yoghurt. |
|  | Dietary Acculturation: **Enabler** | 28) In7: That's a big change for me after coming here. I never used to eat any of those things back home. But after coming here only, I started eating that, and nowadays maybe it's once in two weeks, sometimes we buy Domino's, or very rarely we go to KFC. Most of the time it's to Domino's and Subway. |
|  | Affordability: **Enabler** | 29) In9: When you come to a new country, you think about the cost maximization and minimization. And that's the reason we focus on KFC and McDonald's because of the price, the reasonable price. |
| **4. Increased consumption of cakes, biscuits, chips and snacks (CBCS)** | Substitute for traditional sweets: **Enabler** | 30) FGDP7: All festival foods (traditional sweets) consumption is reduced but stuff like cake and you know, we like cupcakes and ice cream.  31) FGD P13: You get less traditional sweets here, you substitute with chocolates and other stuff that you get more here. |
|  | Dietary acculturation: **Enabler** | 32) In 1: We are eating a lot of crisps here. When we were back in our country we used to eat kind of fruits and stuff like that, but here when we are getting crisps so easily and in a lot of variety of flavors, so we are eating lots of that.  33) In 8: That (consuming CBCS) deviates your diet from what you used to have in India. I never used to have so much of chips, I never used to have so much of biscuits, that sweet biscuits, and the cereals as well. |
|  | Accessibility and affordability: **Enablers** | 34) In1: Bakery food as well here, I think that there is a lot of variety that is easily available, stuff that we didn't have there (home country), so I think that's quite attractive…..the sugary foods and salty foods are getting too much in our lives now.  35) In6: I started eating these cookies. Generally butter cookies, ….. yeah. here, I started consuming. chips, and I started eating lot of the Snickers chocolates. This consumption has increased. Which, I'm (was) not very fond back home.  36) In8: For example, chips and biscuits, so you see Tim Tam is so tempting, so you tend to buy when it is in offer you buy more. So, definitely sugar intake is increased. That deviates your diet from what you used to have in India. I never used to have so much of chips, I never used to have so much of biscuits, that sweet biscuits. |
| **5. Physical Activity** | Cold weather: **Barrier** | 37) FGD P16: Weather is not suitable (for being physically active).  38) In6: My major concern here, only one thing I find it is the cold weather, which is I'm still getting adjusted to the weather. So, as it's very cold, I try my best not to step out walking. |
|  | Time constraints due to work or family responsibilities: **Barrier** | 39)In 6: it's been I think it's now three years before three years I used to regularly go to the gym. But after my daughter, my life has been very busy …and I didn't get time for gym-ing.  40) In 9: if my job changes, I would certainly join the gym or do a regular activity, like do exercise maybe at home or walk or run after my work. |
|  | Lack of peer support: **Barrier** | 41)In 4: I want company. I think that's my problem. I think (walking) anywhere is fine as long as I have some company.  42)In 5: I would actually prefer to have peers, to have some friends. That would be kind of a motivation. You would keep on talking…., while you do the physical activity, ..that will benefit both of you (us). |
|  | Cultural differences: **Barrier** | 43) In 8: I am interested (to be part of a group) but there was one incident when I was jogging, going for work all alone, by myself, there was one community, like the runner’s community …they asked me to join but I did not go…when we meet a new person and say hello on the way, but we might not join them for a talk while you are walking with them. .. sometimes we think how they might think when you ask (talk to) them... that`s cultural barrier. |
|  | High cost of recreational sports: **Barrier** | 44) In3: I would like to do swimming, but one is you have to spend money. The cost is quite high, per session you have to spend $7 or something… we did indoor football, in the winters also. But we had to pay that and everybody in our community were not willing to pay that. That's the main reason we stopped playing for (in) the winter. |

Supplementary Table 2: Solutions for addressing unhealthy dietary and activity behaviours

| **Theme** | **Codes associated with the theme** | **Illustrative Quotes** |
| --- | --- | --- |
| 1. Inadequate consumption of fruits, vegetables, and lentils | Using locally available fruits, vegetables, and lentils | 1. FGD P15: I think the first thing is you have to accept it. These are the options available (in NZ) that’s it. You have to just accept it. 2. FGD P7: With respect to fruits. I think the variety is different here. But I wouldn’t agree that we have only one or two fruits here. Because like the kiwi fruit is so common, which is really good. Yeah, So I mean, given the plums and varieties of apples, apples are very fresh. And if we have those every day, it’s very good for health. 3. FGD P10: I like apples and pears from the farmers market you get a variety of apples. Okay, apples are very costly in India basically you don`t get that much that good fruit in India. But here, Apple and Kiwi are the two things which you can afford. 4. FGD P9: So in fact I am saying that .. I have increased my consumption of green leafy vegetables after coming to NZ. Because I buy those salads and the different types of mesclun and all these leafy leaves Yeah, and baby spinach and then I make salads every night and eat whereas back at home I never ate green leafy vegetables. We used to see the way they were grown in just outside railway tracks. Yeah, so never had those. 5. In 6: Well, that is, now we are getting used to this new... Broccoli is not something which we don't have, back home we don't eat much but here, we take (eat) lot of broccoli because it has more proteins and everything. So, we have started adding those new veggies in out diet. 6. In 7:….. for leafy veggies we have started using silverweed(beet) and kale 7. FGDP5: Compared to India after coming here we are having more of different varieties (of lentils) before coming here, we eat only toor dhal (pigeon peas) after coming here we are trying all dhals. Maybe I am eating more what used compare back in India. Probably because of availability of more varieties. |
|  | Substituting local vegetables in South Asian cuisine | 1. FGDP7: we don’t have kind of green here (which we had in India), tried to cook the available greens in similar way, tasted really good. Kale tasted really good, I made pulau and aroma is quite good 2. FGDP3: Providing information of substitute vegetables would be helpful. Yeah. It's like a transition for some time it would be difficult. … But if I get any information that you will be eating the same thing, same nutrients (from locally available vegetables), then I would switch. |
|  | Using frozen and canned traditional vegetables | 1. FGD P1: (one way to increase vegetable consumption is to)…. use frozen foods 2. FGDP2: … western people eat a lot of canned readymade food, instantly available (for cooking) 3. FGD P14:….people should be aware they (canned lentils) are easy to cook like 30 minutes (for cooking a meal with lentils), it's easy and filling. |
|  | Overcoming difficulty in cooking dry lentils | 1. FGDP12: And also, some people I hear that it's hard to cook (lentils) maybe without the pressure cooker. I told her (my friend) it's very easy to cook in the pressure cooker just five to 10 minutes it will be ready maybe it's one of the reason people are not eating enough because not easy to cook. |
|  | Education on the nutrition quality of canned lentils | 1. In 9: Yeah, that (nutrition information of canned lentils) would certainly be helpful because sometimes what happens when I look at a product and if I don't exactly know what is (in) it. And that's why I am scared of buying it. But if I would certainly have proper information…, then I would not be scared of going out and buying it |
|  | Growing your own to reduce the cost of vegetables | 1. FGDP2: You can grow your own vegetables in your backyard. 2. In2: Yes, I grew methi, (fenugreek) leaves. Yeah, and spinach also, I did. I did some potatoes. Yeah. Yeah, it was good. It depends on the season when you do that. When it is a right season, it comes nicely. I've also tried to do coriander. Mint is very good here. It spreads, and it also gives nice mint leaves. 3. In8: Yes, now, this is the season I'm starting with some garlic seeds that have grown from the previous year and I planted some potatoes, and some leafy vegetables. |
|  | Growing vegetables in containers | 1. In5: yes, the information on how to grow these things in containers or maybe rice bags that you buy rice and you have those bags left over. Demonstration videos on growing techniques in above methods would be really awesome. 2. In2: Yeah, pointers, how to grow vegetables in pots, or sacks, or whatever it is that you can do, even if you're living in an apartment, would be helpful. |
| **2. Increased consumption of poultry and meat** | Increasing vegetable and lentil consumption to reduce meat consumption | 1. In3: My thing is if you fix the vegetables it will come up (lentil consumption). It's like we don't have a habit of taking (eating) lentils only because we only know that we have to eat lentils with rice and vegetables. ….If vegetable is fixed then I can fix (lentil consumption), it's a simple thing. …., but vegetables is replaced by meat here. |
| **3. Increased consumption of dairy fats, oils and European takeaways** | Knowledge on optimal portion size of dairy food and oils | 1. FGDP18: The other thing is educating people to stop deep fry anything because that is the killer. 2. In3: If you say cheese is bad for me, that will give a negative effect on me. But if you say like, a one slice of cheese or a gram or whatever you need (recommended), you can say like, okay, if you eat that, that's good for health. But if you increase that, (that might not be good for your health). If you say that then that's good for me. The word (framing the message positively) used for the thing (dairy food) that we are consuming, and we feel like that is good. 3. In 5: I would like to have more information on saturated fat. 4. In8: …. cheese we use for the bread, and I would say at least 500 grams to one kg in a month. Yeah and the ghee. Yeah, how much is ideal. If you can provide the information on how much fats are good for us. That will definitely be useful. |
| **4. Increased consumption of cakes, biscuits, chips and snacks (CBCS)** | Increase awareness and knowledge on reading and interpreting nutritional labels and optimal portion sizes | 1. FGDP4: yeah that kind of information (nutrition labels) is really important. Especially because people from India for example, based on my experience, we get everything from family and our mother would take care of us. So we do not know about nutrients and what a serving should look like 2. In 1: Raw quantity or exact amount of fat in it, no, not of that exactly so I think, when we are buying, I don't think that we are even exactly thinking about that (fat) at that moment. 3. In 2: Yeah, I know some information, but I don't know the exact, this much grams or this much fats are there in that. Maybe after seeing the container, we'll get to know, "Oh, there's lot of sugar in this. There's lot of fat in it." |
| **5. Physical Activity** | Ways to increase physical activity | 1. FGD P3: So also, we have to getting to know your city (by going for walks), there are so many beautiful trails. You can do with your family, friends. 2. In 7: That's something good in Dunedin. I've got a colleague of mine who took us for a trekking in Harrington point. That was a real good experience, and we are planning to do something (similar) soon. 3. In 4: Put together a walking group or something like that. you know, I mean having our community would be somewhat better because we can just enjoy more fun talks (as we walk).. It will be great (if you provide walking trails information) because I really don't know about walking trails. 4. In 1: I think that if there is a group of parents or if the people in your area are interested (group walking)…or there are people around that are in the school or the school would promote something like that so I think that would be a good motivation. |
|  | Preferred types of activities | 1. In5: They (preferred activities) would be brisk walking maybe, then the dance. 2. In 6: Yeah, even when I joined with my Indian community so we just gather up and we put some music and we play and we dance, which really love to do. 3. In 4: That would be lovely having an Indian dance club! And something to do in (a) group is always so much more interesting than doing just alone at home. 4. In 3: ….but in our case …, like we had a Nepalese community and we started playing football. 5. In8: I do have some friends here with whom I go for walks, sometimes I go play cricket or tennis. So you can socialize and also you can out some body workout. |
